# Supplementary material for: Association of helicobacter pylori infection with lipid metabolism and 10-year cardiovascular risk in diabetes mellitus: A cross-sectional study
Source: PLoS One. 2025 Apr 9;20(4):e0319688. doi: 10.1371/journal.pone.0319688 (PMC11981135; doi:10.1371/journal.pone.0319688)
Supplement: Attachment — Submitted filename: Reply to reviewers 20250109.docx [file pone.0319688.s004.docx]

PONE-D-24-47046

Association of H. pylori infection with lipid metabolism and 10-year cardiovascular risk in diabetes mellitus: a cross-sectional Study

Dear Editor and Reviewers,

Many thanks for your detailed and thoughtful review comments. We have correspondingly revise the manuscript and wrote this reply to reviewer letter.

Reply to Editor:
==============================
The manuscript provides valuable insights into the link between H. pylori infection, lipid metabolism, and cardiovascular risk in diabetes. However, major revisions are needed to improve clarity and rigor:

Introduction: The rationale for the study requires expansion, with a stronger focus on addressing gaps in the literature and a more cohesive narrative.

**Response:** Thank you for your valuable feedback. In the revised introduction, we have expanded the rationale for the study by addressing the gaps in the literature more comprehensively. Specifically, we have elaborated on the systemic effects of *Helicobacter pylori* (HP) infection, particularly its influence on lipid metabolism and cardiovascular risk, with a focus on diabetic populations. Additionally, we have restructured the introduction to present a more cohesive narrative by integrating the connections between HP, dyslipidemia, diabetes, and cardiovascular disease. These changes aim to provide a clearer and more compelling justification for the study.

Methodology: The authors should provide greater detail on inclusion criteria, confounding variables, diagnostic methods, and cardiovascular risk assessment tools. Clarify retrospective data collection processes and address potential biases.

**Response:** We have carefully revised the Materials and Methods section to address your summary comments as follows:

1. **Inclusion Criteria & Study Population:**
   - Expanded the description of inclusion and exclusion criteria.
   - Added a CONSORT flowchart (Figure 1) to illustrate participant recruitment.
2. **Confounding Variables:**
   - Included additional variables such as diabetes duration, HbA1C, treatment for diabetes and dyslipidemia, and GFR where available.
   - Clarified limitations regarding unavailable data and adjusted for key confounders like age, sex, and exercise in our analysis.
3. **Diagnostic Methods:**
   - Provided a detailed explanation of the 13C-urea breath test (13C-UBT) for H. pylori detection, including the rationale for its use and validation of the DPB threshold with citations.
   - Discussed the limitation of not using serological tests for past infections.
4. **Cardiovascular Risk Assessment Tools:**
   - Elaborated on the use of the Framingham Risk Score (FRS), its limitations in diabetic populations, and justified its selection over alternative tools.
   - Discussed the validity of risk thresholds used for our diabetic cohort.
5. **Retrospective Data Collection & Potential Biases:**
   - Clarified the retrospective data collection process, including data sources and timeframes.
   - Addressed potential biases by detailing mitigation strategies, such as using validated questionnaires and standardized measurement protocols.

We believe these revisions enhance the clarity and robustness of our methodology. The changes have been highlighted in the revised manuscript for your review.

Results: Tables need clearer formatting, consistent terminology, and precise reporting of significant findings.
**Response:** Revised as suggested. Format of all tables from 1 to 4 has been improved. Terminology and corresponding abbreviations were corrected for consistency. Bolding has been applied to all significant p-values (p < 0.05) for improved visibility.

Discussion: Emphasize the study’s cross-sectional nature and avoid causal inferences. Improve thematic organization and provide balanced interpretations of proposed mechanisms.

**Response:** Thank you for your thoughtful review of our manuscript and for your valuable suggestions. We appreciate your advice on emphasizing the study's cross-sectional nature, avoiding causal inferences, improving thematic organization, and providing balanced interpretations of proposed mechanisms.

In response to your recommendations, we have made the following revisions:

1. **Emphasizing Cross-Sectional Nature and Avoiding Causal Inferences:**

- **Language Adjustments:** We have carefully reviewed the manuscript to eliminate any language implying causation. We replaced phrases such as "HP infection is an independent risk factor" with "HP infection is independently associated with," to reflect the associative nature of our findings.
- **Acknowledgment of Study Limitations:** We have prominently acknowledged the limitations of our cross-sectional study design in both the Discussion and Conclusion sections: *"As a cross-sectional investigation, it can only demonstrate the correlation between HP infection and abnormalities of lipid metabolism and cannot clarify the causation between them."* *"However, the cross-sectional nature of this study limits the ability to infer causation, and further longitudinal studies are warranted to confirm these findings."*

2. **Improving Thematic Organization:**

We reorganized the Discussion section to enhance thematic coherence. The Discussion now follows a logical progression:

- **Summary of Main Findings:** We begin by summarizing the key results, highlighting the associations observed.
- **Possible Mechanisms:** We delve into potential mechanisms explaining the association between HP infection and dyslipidemia in patients with diabetes, discussing inflammation, insulin resistance, hepatic function, and gut microbiota alterations.
- **Analysis of Specific Findings:** We address specific results, such as the lack of significant difference in triglyceride levels, providing possible explanations.
- **Inconsistencies in Literature:** We discuss inconsistencies in the literature regarding HP infection severity and its impact on dyslipidemia, acknowledging differing findings and suggesting areas for future research.
- **Strengths and Limitations:** We outline the strengths of our study and provide a detailed discussion of its limitations, including the acknowledgment of missing key variables and limitations inherent in our physical activity assessments.

3. **Providing Balanced Interpretations of Proposed Mechanisms:**

- **Balanced Discussion:** We have revised the sections discussing potential mechanisms to provide a balanced interpretation, incorporating evidence that both supports and challenges our hypotheses. For example, while exploring the role of inflammation, we acknowledge that:*"Factors contributing to these inconsistencies may include differences in study populations, variations in bacterial strains, genetic predispositions of hosts, and environmental influences such as diet and lifestyle."*
- **Citing Relevant Studies:** We have added references to studies that offer alternative perspectives, ensuring that readers are presented with a comprehensive view of the current research landscape.

We believe these revisions have significantly improved the thematic organization of the Discussion and provided a more balanced interpretation of the proposed mechanisms. By emphasizing the cross-sectional nature of our study and avoiding causal inferences, we aim to present our findings responsibly and accurately.

Ethics and Transparency: Include missing ethics details, address discrepancies in data collection, and provide clarity on handling “unreliable” data.

**Response:** The suggested points have been accepted for revising the corresponding manuscript’s part and corresponding reply has been made to each reviewer’s comment.

Language: Revise for grammatical accuracy, consistent use of abbreviations, and appropriate terminology.
**Response:** The whole manuscript has been now polished for grammatical accuracy, consistent use of abbreviations, and appropriate terminology.

Journal requirements:

When submitting your revision, we need you to address these additional requirements.

1. Please ensure that your manuscript meets PLOS ONE's style requirements, including those for file naming. The PLOS ONE style templates can be found at https://journals.plos.org/plosone/s/file?id=wjVg/PLOSOne_formatting_sample_main_body.pdf and https://journals.plos.org/plosone/s/file?id=ba62/PLOSOne_formatting_sample_title_authors_affiliations.pdf

**Response:** The formatting has been corrected by following the provided documents.

2. Please note that funding information should not appear in any section or other areas of your manuscript. We will only publish funding information present in the Funding Statement section of the online submission form. Please remove any funding-related text from the manuscript.

**Response:** The funding section is now removed in the end of manuscript.

3. Thank you for stating the following financial disclosure: “This study was supported by the scientific research program of the Sichuan Medical and Health Care Promotion Association (Project No. KY2022SJ0100).”

Please state what role the funders took in the study. If the funders had no role, please state: "The funders had no role in study design, data collection and analysis, decision to publish, or preparation of the manuscript." If this statement is not correct you must amend it as needed. Please include this amended Role of Funder statement in your cover letter; we will change the online submission form on your behalf.

**Response:** The Role of Funder statement is now added in the cover letter, as below: ”This study was supported by the scientific research program of the Sichuan Medical and Health Care Promotion Association (Project No. KY2022SJ0100). The funders had no role in study design, data collection and analysis, decision to publish, or preparation of the manuscript."

4. We note that your Data Availability Statement is currently as follows: “All relevant data are within the manuscript and in Supporting Information files.”

Please confirm at this time whether or not your submission contains all raw data required to replicate the results of your study. Authors must share the “minimal data set” for their submission. PLOS defines the minimal data set to consist of the data required to replicate all study findings reported in the article, as well as related metadata and methods (https://journals.plos.org/plosone/s/data-availability#loc-minimal-data-set-definition). For example, authors should submit the following data: - The values behind the means, standard deviations and other measures reported; - The values used to build graphs; - The points extracted from images for analysis. Authors do not need to submit their entire data set if only a portion of the data was used in the reported study. If your submission does not contain these data, please either upload them as Supporting Information files or deposit them to a stable, public repository and provide us with the relevant URLs, DOIs, or accession numbers. For a list of recommended repositories, please see https://journals.plos.org/plosone/s/recommended-repositories. If there are ethical or legal restrictions on sharing a de-identified data set, please explain them in detail (e.g., data contain potentially sensitive information, data are owned by a third-party organization, etc.) and who has imposed them (e.g., an ethics committee). Please also provide contact information for a data access committee, ethics committee, or other institutional body to which data requests may be sent. If data are owned by a third party, please indicate how others may request data access.

5. When completing the data availability statement of the submission form, you indicated that you will make your data available on acceptance. We strongly recommend all authors decide on a data sharing plan before acceptance, as the process can be lengthy and hold up publication timelines. Please note that, though access restrictions are acceptable now, your entire data will need to be made freely accessible if your manuscript is accepted for publication. This policy applies to all data except where public deposition would breach compliance with the protocol approved by your research ethics board. If you are unable to adhere to our open data policy, please kindly revise your statement to explain your reasoning and we will seek the editor's input on an exemption. Please be assured that, once you have provided your new statement, the assessment of your exemption will not hold up the peer review process.

6. Your ethics statement should only appear in the Methods section of your manuscript. If your ethics statement is written in any section besides the Methods, please delete it from any other section.

**Response:** The ethics statement has been integrated into the Methods section and removed in the end of manuscript.

7. Please include a separate caption for each figure in your manuscript.

**Response:** Our manuscript includes only one figure, Figure 1, which illustrates the participant recruitment process. We have ensured that this figure has a separate and appropriately detailed caption placed directly below the figure, adhering to the journal’s

Reviewers' comments:

Reviewer's Responses to Questions

**Comments to the Author**

1. Is the manuscript technically sound, and do the data support the conclusions?

The manuscript must describe a technically sound piece of scientific research with data that supports the conclusions. Experiments must have been conducted rigorously, with appropriate controls, replication, and sample sizes. The conclusions must be drawn appropriately based on the data presented.

Reviewer #1: Yes

Reviewer #2: No

Reviewer #3: Yes

Reviewer #4: Yes

Reviewer #5: No

2. Has the statistical analysis been performed appropriately and rigorously?

Reviewer #1: Yes

Reviewer #2: Yes

Reviewer #3: Yes

Reviewer #4: Yes

Reviewer #5: Yes

3. Have the authors made all data underlying the findings in their manuscript fully available?

The PLOS Data policy requires authors to make all data underlying the findings described in their manuscript fully available without restriction, with rare exception (please refer to the Data Availability Statement in the manuscript PDF file). The data should be provided as part of the manuscript or its supporting information, or deposited to a public repository. For example, in addition to summary statistics, the data points behind means, medians and variance measures should be available. If there are restrictions on publicly sharing data—e.g. participant privacy or use of data from a third party—those must be specified.

Reviewer #1: Yes

Reviewer #2: Yes

Reviewer #3: Yes

Reviewer #4: Yes

Reviewer #5: Yes

4. Is the manuscript presented in an intelligible fashion and written in standard English?

PLOS ONE does not copyedit accepted manuscripts, so the language in submitted articles must be clear, correct, and unambiguous. Any typographical or grammatical errors should be corrected at revision, so please note any specific errors here.

Reviewer #1: No

Reviewer #2: Yes

Reviewer #3: Yes

Reviewer #4: Yes

Reviewer #5: Yes

5. Review Comments to the Author

Please use the space provided to explain your answers to the questions above. You may also include additional comments for the author, including concerns about dual publication, research ethics, or publication ethics. (Please upload your review as an attachment if it exceeds 20,000 characters)

Reviewer #1: I thank the editor for sending me this manuscript for review.
1. In this paper, the authors aimed to investigate the effect of HP infection on the risk of lipid metabolism disorders and cardiovascular disease in patients with diabetes mellitus.
2. First, I advise the authors next time they submit the paper to include the continuous line count, to make the review more accurate and timelier.

**Response:** The continuous line count is added as suggested.
3. It is also important for authors to make a linguistic and grammatical revision to make the manuscript more discursive, especially in the first part of the paper.

**Response:** We have thoroughly reviewed and revised the manuscript to enhance its linguistic quality and grammatical accuracy, with particular attention to the first section. These revisions aim to improve the overall clarity, coherence, and readability of the manuscript.

4. In general, I find an adequate work, well-structured and constructed, with good description of the moments and steps taken. A revision is needed to better clarify passages in the introduction and in the materials and methods, but it is certainly a doable job on the part of the authors.
**Response:** Thank you for your positive feedback and constructive suggestions. We appreciate your recognition of the structure and clarity of our work. In response to your recommendation, we have thoroughly revised the **Introduction** and **Materials and Methods** sections to enhance clarity and ensure that all passages are well-explained and easily understandable.

Below the authors will find timely comments on the various parts of the manuscript

Abstracts and Keywords
5. I advise the authors to remove as many abbreviations as possible to make this section of the manuscript as fluent and discursive as possible to facilitate the reading and fluency of the abstract itself

**Response:** Thank you for your valuable feedback. We agree that minimizing abbreviations can enhance the readability and fluency of the abstract. In response to your suggestion, we have revised the abstract to reduce the number of abbreviations, ensuring that the content remains clear and accessible to a broader audience. Abbreviations that are essential for clarity and commonly recognized have been retained, while others have been expanded or removed as appropriate.
6. Appropriate and relevant keywords, if I were the authors I would look for perhaps 2 or 3 additional keywords, to increase the possible indexing of the manuscript and facilitate searches associated with it. I recommend making use of the MESH database for this <https://www.ncbi.nlm.nih.gov/mesh/>

**Response:** Thank you for your valuable suggestion. We have reviewed and expanded our list of keywords to include additional terms from the Medical Subject Headings (MeSH) database. This enhancement aims to improve the manuscript's indexing and facilitate more effective searches. The revised list includes two additional relevant keywords that align with the study's focus and are commonly used in related research.

Introduction
7. This section of the paper should be a bit more developed by the authors.

**Response:** Thank you for your suggestion. In the revised introduction, we have significantly expanded the content to provide a more detailed and comprehensive discussion of the study's background. For example, we have elaborated on the systemic impact of HP infection, its association with dyslipidemia, and its implications for cardiovascular risk, particularly in diabetic populations. These additions aim to address the need for a more developed introduction.
8. Corrected the beginnings of the topics covered but subsequently the authors were a bit too concise and schematic. I recommend more development of both the part where they describe HP, then describe lipids and related issues, cardiovascular issues, and finally the part where they talk about the correlation between HP and diabetes.

**Response:** We appreciate your detailed feedback. In response, we have expanded each section of the introduction to provide a more thorough discussion. For example:

- The description of HP now includes its role in both gastrointestinal and non-gastrointestinal diseases, with specific examples and references to its systemic effects.

- The discussion of lipids and cardiovascular issues has been expanded to include the role of dyslipidemia as a risk factor for cardiovascular diseases and the specific impact of HP infection on lipid profiles.

- The correlation between HP and diabetes has been further developed, emphasizing the bidirectional relationship between these conditions and the potential impact of HP on systemic inflammation, oxidative stress, and lipid metabolism in diabetic patients.

9. I advise the authors to try to make “connections” between the various topics so that the introduction will be somewhat discursive and not telegraphic whenever the topic under analysis is changed.

**Response:** Thank you for this important suggestion. In the revised introduction, we have improved the logical flow and coherence by creating stronger connections between the various topics. For instance, we now transition more smoothly from the discussion of HP's systemic effects to its specific impact on dyslipidemia and cardiovascular risk, and then to its relevance in diabetic populations. These changes aim to make the introduction more discursive and less schematic.

Materials and methods
Study population
10. In this section the authors should not include numbers or anything else but should describe the inclusion and exclusion criteria of the study. The numbers of those included and excluded and the reasons will then be included in the results, right at the beginning.

**Response:** We have revised the Methods section to include detailed descriptions of inclusion and exclusion criteria. Numerical data on excluded participants have been moved to the Results section.

11. An additional thing to develop at this point is a flowchart (CONSORT) with the just that described (Inclusion and exclusion and at the end the number of patients enrolled). I recommend looking in this link.
<https://www.equator-network.org/reporting-guidelines/consort/>

**Response:** A CONSORT flowchart illustrating inclusion and exclusion criteria and final enrollment numbers has been added as Figure 1.

Figure 1. Graphical flowchart for the participant recruitment process

Discussion
12. Adequate and well argued. Nothing to say.

**Response:** Thanks for your approvement on the Discussion section.

Reviewer #2: The study evaluated the relationship between H. pylori infection and dyslipidemia in 266 patients with diabetes and suggested a possible increased risk of CVDs in H. pylori-positive patients. The result supported the potential effect of H. pylori on total cholesterol, LDL, and HDL.
The study method is sound. However, the presentation in the discussion needs to be improved significantly, and the topic has been explored using larger and cohort studies (PMID: 39054452 and PMID: 36974892) The current uncertainties are mainly focused on how the disease severity could change both dyslipidemia and its change when eradicated which are not addressed in the present report. There are some other points that I think will improve the study:

1. Both in the conclusion section of the abstract and the discussion section, I suggest modifying the definitive sentences like “H. pylori infection is a risk factor for dyslipidemia”, as the cross-sectional nature of the study prevents drawing strong evidence.

**Response:** We agree that the cross-sectional design of our study precludes establishing causation and that the use of definitive language may overstate our findings. To address this concern, we have revised the conclusion in both the abstract and the discussion sections to reflect the association rather than causation. The revised text now reads as follows:

- **Abstract Conclusion:** "H. pylori infection is associated with dyslipidemia and may contribute to an increased risk of cardiovascular disease in diabetic patients."
- **Discussion Conclusion:** "Concurrent H. pylori infection is associated with dyslipidemia in individuals with diabetes. Compared to diabetic patients without H. pylori infection, those with concurrent infection exhibit elevated FRS and a potentially increased 10-year risk of cardiovascular diseases. However, the cross-sectional nature of this study limits the ability to infer causation, and further longitudinal studies are warranted to confirm these findings."

We believe these changes appropriately address your concern and ensure that the conclusions are consistent with the study's design and limitations. Thank you for helping us improve the clarity and accuracy of our manuscript.

2. Proton pump inhibitors could effect the urea breath tests. Was this considered when conducting the test? If not, it should be mentioned as a limitation.

**Response:** We appreciate the reviewer’s insightful comment regarding the potential influence of proton pump inhibitors (PPIs) on the 13C-urea breath test (13C-UBT) results. To address this concern, we have clarified in the manuscript that participants were instructed to avoid medications, including PPIs, that could interfere with the test results.

**Revisions made in the manuscript:** We have updated the "HP detection" section to explicitly state that participants were confirmed to have abstained from PPIs, antibiotics, and gastric mucosal protectants for at least one month prior to the test. The revised text now reads as follows:

**Revised Text in Manuscript:** "The 13C-urea breath test (13C-UBT) was used to predict whether participants were infected with *H. pylori*. All participants underwent the 13C-UBT on an empty stomach in the morning. The Delta Over Baseline (DOB) of 13C-UBT determines whether the participant is infected with *H. pylori*. DOB values ≥4 indicated the *H. pylori*-infected group (DMHP+ group), whereas DOB values <4 indicated the non-*H. pylori*-infected group (DMHP- group). Prior to testing, it was confirmed that participants had not taken antibiotics, gastric mucosal protectants, or proton pump inhibitors (PPIs) within the past one month to avoid potential interference with the test results."

3. In the method section, Besides the factors that have already been adjusted for, there are other important variables that I highly recommend adding: diabetes duration, HbA1C, treatment for diabetes and dyslipidemia and GFR.
Also, for comparing CVD risk, I suggest adjusting for diabetes duration, HbA1C, and exercise.

**Response:** We appreciate the reviewer’s insightful suggestions regarding the inclusion of additional variables in the analysis. We acknowledge the importance of these variables in understanding the relationships explored in our study. However, we encountered the following limitations with our dataset, which made it infeasible to include some of the suggested variables:

1. **Diabetes Duration:** Unfortunately, the physical examination questionnaire only included a binary variable indicating a history of diabetes, without an option to specify diabetes duration. Therefore, we do not have data on diabetes duration for the study participants.
2. **HbA1C and GFR:** These tests were voluntary during the physical examination, and as a result, we do not have complete data for glycated hemoglobin (HbA1C) and glomerular filtration rate (GFR). The incomplete data would introduce significant bias if included in the analysis.
3. **Treatment for Diabetes and Dyslipidemia:** Since this study was based on data collected during a physical examination, there was no documentation of participants’ treatment regimens or medication history. Thus, we are unable to include treatment-related variables in the analysis.
4. **Exercise:** Exercise data was collected via the International Physical Activity Questionnaire (IPAQ) short-form survey. The total intensity of participants' physical activities over the past seven days was calculated in terms of metabolic equivalents (METs). These were categorized into high-intensity, moderate-intensity, and low-intensity groups, and this variable was already included in our statistical analysis. The details of this categorization are described in the Methods section, and the results are presented in Table 1. We found statistically significant differences in exercise status between the DMHP- and DMHP+ groups (P < 0.05), and exercise was adjusted for in the multivariate logistic regression models.

While we were unable to include diabetes duration, HbA1C, GFR, and treatment variables due to the limitations outlined above, we have ensured that the analysis accounts for other relevant confounding factors such as age, sex, and exercise. We have revised the Methods section to clarify these limitations and provide an additional discussion in the Limitation part of Discussion section.

4. What were the criteria for diabetes? Please include in the method section, were all patients type 2?

**Response:** Thank you for your comment. Yes, all patients included in the study were diagnosed with Type 2 diabetes mellitus.

To clarify, the inclusion criteria for the study have been detailed in the Methods section. Specifically, the study focused on patients with Type 2 diabetes mellitus who met the following criteria: willingness to fully participate, availability of reliable laboratory results, and completion of all required measurements and questionnaires. Exclusion criteria included individuals who declined certain measurements, had incomplete questionnaires, or were diagnosed with conditions such as tumors, liver diseases (e.g., hepatitis, cirrhosis, or schistosomiasis-related liver disease), or presented with unreliable laboratory results. These measures ensured a well-defined and representative sample of eligible participants, as illustrated in Figure 1 of the manuscript.

5. In Table 2, please change 0 values to “reference” or an equivalent. Additionally, bolding the significant p-values and modifying the values for distinguishing ß from ORs help with the readability of the table.

**Response:** We appreciate the reviewer’s valuable suggestion to improve the clarity and readability of Table 2. In response, we have made the following revisions:

- Replaced all "0" values in the table with “Reference” to clarify the baseline/reference groups for each categorical variable.
- Bolding has been applied to all significant p-values (p < 0.05) to enhance visibility and readability.
- We have clearly distinguished β coefficients (used for continuous variables) from Odds Ratios (ORs) (used for categorical variables) by underlining ORs in the table and grouping either continuous variables or categorial variables together. A corresponding explanatory note has been added below the table to define these terms and guide interpretation.
- A detailed note has been added under the table explaining the use of β coefficients, ORs, and the concept of “Reference” for categorical variables, as well as the emphasis on bolded significant p-values.

We believe these revisions address the reviewer’s concerns and improve the clarity, interpretability, and overall quality of Table 2.

6. "Diabetic patients" is a non-optimal term. I suggest changing it to patients with diabetes across the manuscript globally.

**Response:** Thank you for bringing this to our attention. We agree that using person-first language is important to emphasize the individual rather than the condition. Throughout the manuscript, we have replaced the term "diabetic patients" and “diabetes patients” with "patients with diabetes" to reflect this preference and ensure respectful and patient-centered terminology.

7. The second paragraph of the discussion is mainly devoted to providing insights into how dyslipidemia contributes to CVD. It is not the main focus of the study and is too long for the current title. Additionally, the talk on how HP can contribute to dyslipidemia is in the same paragraph, making it too long. I suggest bringing the HP mechanisms as the second paragraph, separating the two, and only a brief mention of the corresponding mechanisms of dyslipidemia in CVD. There are other subjects in the same paragraph as well; I suggest separating them into other paragraphs, too.

**Response:** Thank you for your insightful feedback on the organization of the Discussion section. We agree that the second paragraph was too lengthy and covered multiple topics, which may detract from the main focus of our study. In response to your suggestions, we have revised the Discussion to improve clarity and alignment with our study objectives.

Specifically, we have:

1. **Reorganized the Paragraphs:**
   - We have separated the original second paragraph into multiple, shorter paragraphs, each focusing on a specific topic. This reorganization enhances readability and ensures that each paragraph addresses a single idea.
2. **Focused the Second Paragraph on H. pylori Mechanisms:**
   - We have moved the discussion on the mechanisms by which *Helicobacter pylori* (H. pylori) infection may contribute to dyslipidemia to the second paragraph, immediately following the presentation of our main findings in the first paragraph. This aligns the content with the primary focus of our study.
3. **Provided a Brief Mention of Dyslipidemia in CVD:**
   - We have included a concise overview of how dyslipidemia contributes to cardiovascular disease in a separate, subsequent paragraph. This ensures that the discussion remains relevant without overshadowing the main topic.
4. **Separated Other Subjects into Individual Paragraphs:**
   - Additional topics previously included in the same paragraph, such as the detection of H. pylori DNA in atherosclerotic plaques, the role of overall infection burden, and the impact of high-salt diets, have been separated into their own paragraphs. This allows for clearer and more focused discussions of each point.
5. **Ensured Smooth Transitions Between Paragraphs:**
   - We carefully reviewed the transitions between the first, second, and third paragraphs to maintain a coherent narrative throughout the Discussion section.

8. I suggest expanding on the proposed mechanisms responsible for dyslipidemia in HP, with consideration of inter-related dyslipidemia and diabetes mechanisms. Explaining why TG was not affected, but other indicators were is another suggestion. Moreover, currently, there are some inconsistencies regarding how the severity of HP may affect its association with dyslipidemia. I suggest highlighting them.

**Response:**

Thank you for your insightful comments and suggestions, which have helped us to improve and clarify our Discussion section.

**1. Expanded Mechanisms Responsible for Dyslipidemia in H. pylori Infection:**

We have expanded our discussion on the mechanisms by which H. pylori infection may lead to dyslipidemia, particularly in the context of diabetes. We have elaborated on how H. pylori-induced systemic inflammation can exacerbate insulin resistance, which is closely linked to dysregulation of lipid metabolism. We also discussed the potential impact of H. pylori on hepatic function and gut microbiota composition, highlighting the complex interplay between these factors in contributing to dyslipidemia.

**2. Explanation for Unaffected TG Levels:**

We have added a dedicated paragraph addressing why triglyceride levels were not significantly affected in our study. We propose that H. pylori infection may have a more selective effect on cholesterol metabolism rather than on triglyceride synthesis and regulation. We also consider the influence of diabetes-related factors and medications that might modulate TG levels independently of H. pylori infection.

**3. Highlighted Inconsistencies Regarding the Severity of H. pylori Infection:**

We have included a discussion on the inconsistencies in the literature concerning the relationship between the severity of H. pylori infection and dyslipidemia. We examined studies with varying findings and explored potential reasons for these discrepancies, such as differences in bacterial strains, host factors, and methodological approaches. We acknowledged that our study did not assess infection severity or strain-specific characteristics, suggesting that future research should address these aspects to provide a clearer understanding.

We believe these revisions have strengthened our manuscript by providing a more comprehensive and nuanced discussion of the mechanisms involved and addressing the specific points you raised.

9. Please include the ethics committee reference number in the related method section.

**Response:** We appreciate your comment regarding the inclusion of the ethics committee reference number. However, as the Ethics Committee of Deyang People's Hospital waived the requirement for informed consent for this study because the study was a retrospective study utilizing medical examination records on file for medical examination clients. As no formal ethics approval was required, no ethics committee reference number was issued.

Reviewer #3: Thanks for inviting me to review the manuscript entitled Association of H. pylori infection with lipid metabolism and 10-year cardiovascular riskin diabetes mellitus: a cross-sectional Study by li yuexi et al
It is interesting contemporary topic I have gone through the manuscript and I have some problem with understanding the methodology of the study
As per authors it is a retrospective study, the data was retrieved from patients records who attended the Health Management Center of Deyang People's Hospital. As I understand these patients had their regular health check up for Diabetes on routine basis hence such an elaborate monitoring for diabetes usually not taken place ( what I gather from the data)
The authors clearly mentioned that an ethical committee waiver for informed consent was obtained as no direct contact was with patient for the study purpose
Iin this situation I have following comments
1.When and how the response for questionaries were obtained .

**Response:** Thank you for pointing out the need for clarification regarding the timing and method of questionnaire data collection. The questionnaires used in this study were part of the routine health checkup process at the Health Management Center of Deyang People's Hospital. The data were collected at the time of the participants' medical examinations, which were conducted between January 1 and May 31, 2024. These questionnaires were administered as part of the standard protocol for collecting lifestyle information (e.g., tobacco and alcohol consumption, physical activity) during health checkups. As this was a retrospective study, we utilized the archived questionnaire data from the medical records, which were completed by participants at the time of their health checkups. This is now clarified in the revised Methods section (see “Anthropometric, Biochemical, and Lifestyle Data Collection”).

2.Is it possible to get response in a retrospective study answering all specific questions meant for this study

**Response:** We appreciate this insightful question. The retrospective nature of the study relied on pre-existing data collected during routine health checkups. The specific questions used in the study were part of the standardized health questionnaire administered by the Health Management Center. As such, the data were not collected specifically for this study but were already available in the medical records. While retrospective studies have inherent limitations in terms of data collection, the standardized nature of the questionnaires ensured that the information relevant to our study objectives (e.g., smoking and drinking status, physical activity levels) was consistently recorded. This point has been clarified in the revised manuscript to emphasize that the study utilized pre-existing data from standardized questionnaires.

3. whether the questionnaire was validated after a pilot study or not

**Response:** Thank you for raising this important methodological point. The questionnaire used in this study was not specifically designed for the current research but was part of the routine health checkup protocol at the Health Management Center. As such, it was a standardized tool that had been previously validated for use in this population to collect lifestyle and behavioral data (e.g., smoking, alcohol consumption, and physical activity). For example, the International Physical Activity Questionnaire (IPAQ) short form, which was used to assess physical activity levels, is a globally validated tool for measuring physical activity.

4 Is it possible to get all detailed l anthropometric measurements which were mentioned in the study in a retrospective study I want clarification regarding methodology to review the manuscript.

**Response:** We acknowledge your concern about the availability of detailed anthropometric measurements in a retrospective study. The anthropometric data (e.g., height, weight, waist circumference, and blood pressure) were routinely collected as part of the standardized health checkup process at the Health Management Center. These measurements were performed by trained healthcare professionals following standard protocols, and the data were recorded in the participants' medical records. As such, the retrospective study design allowed us to access this detailed information from the archived medical examination records.

Reviewer #4: Thank you for the opportunity to read and review this manuscript. While this study presents valuable findings regarding the potential association between HP infection, lipid metabolism, and CV risk in the diabetic population, there are several suggestions that could further improve the paper:

Introduction, 1) The statement that HP is associated with non-GI diseases is overly vague. To ensure scientific clarity, the authors should define whether this association is positive, negative, or something else.

**Response:** Thank you for pointing this out. In the revised introduction, we have clarified that the association between HP and non-GI diseases can vary, with some studies suggesting a potential negative impact, such as increased systemic inflammation and metabolic disturbances, while others indicate a more complex or neutral relationship. This clarification aims to ensure scientific precision.

Introduction, 2) The authors should elaborate on the rationale for focusing on the diabetic population, as this is a crucial omission. While the potential link between HP and lipid metabolism is acknowledged, the authors haven’t explained why diabetes is particularly relevant to this investigation. Without addressing this, the introduction lacks a key element of the study design rationale.

**Response:** We appreciate this insightful comment. In the revised introduction, we have elaborated on the rationale for focusing on the diabetic population. Specifically, we have highlighted that diabetes is characterized by chronic metabolic disturbances, including dyslipidemia, which significantly elevate cardiovascular risk. Additionally, we have discussed evidence suggesting that diabetic individuals have a higher prevalence of HP infection and lower eradication rates, which may exacerbate systemic inflammation and oxidative stress, further contributing to dyslipidemia and cardiovascular risks. These additions aim to strengthen the study design rationale.

Introduction, 3) The introduction could benefit from improved paragraph structure to enhance the flow and logical progression of ideas in general.

**Response:** Thank you for this suggestion. In the revised introduction, we have restructured the paragraphs to improve the flow and logical progression of ideas. For example, we now begin with a discussion of HP's systemic effects, followed by its specific impact on lipid metabolism and cardiovascular risk, and conclude with its relevance in diabetic populations. This restructuring aims to enhance readability and coherence.

Methods, 1) Although the authors describe the eligibility criteria well, it is unclear why certain conditions, such as tumors and hepatitis, were excluded. The authors should briefly explain the rationale for these exclusions to provide a clearer understanding of the study design.

**Response:** Thank you for pointing this out. We have now clarified the rationale for excluding conditions such as tumors and hepatitis. These conditions were excluded because they can independently influence systemic inflammation and metabolic parameters, which are key variables in our study. This explanation has been added to the subsection Study Population in the Materials and Methods section.

Methods, 2) The use of METs and the IPAQ short-form surveys to estimate exercise intensity is appropriate; however, these methods rely on self-reported data and may introduce misclassification due to subjective biases. The authors should clarify whether any efforts were made to validate these data or, at the very least, address these limitations (such as subjectivity) in the Discussion.

**Response:** We thank the reviewer for pointing out the potential limitations of using self-reported data for estimating exercise intensity. To mitigate recall bias and reduce subjectivity, we made efforts to enhance the accuracy of the data collection process. Specifically, participants were invited to complete the questionnaire together with a family member who could assist in recalling and validating the information provided. We have now clarified this approach in the Methods section (Page X, Paragraph Y) and addressed the residual limitations of self-reported data in the Discussion section (Page Z, Paragraph W).

Methods, 3) A notable gap is the exclusive reliance on the 13C-UBT test. While this test is standard for detecting active HP infections, it does not account for past or inactive infections, which could also influence outcomes, especially in a study assessing long-term risk factors like 10-year CV risk. This limitation may affect the generalizability of the findings. Including serological testing for HP antibodies could have provided complementary data to address this issue.

**Response:** We thank the reviewer for highlighting this important point. We agree that the exclusive reliance on the urea breath test (UBT) may limit the ability to account for past or inactive *Helicobacter pylori* (HP) infections, which could influence long-term outcomes such as 10-year cardiovascular (CV) risk. However, the decision to use the UBT was based on its status as the most recommended diagnostic method for detecting active HP infections, as outlined in the *guideline for primary care of helicobacter pylori infection* at the primary level*.* The UBT directly measures urease activity, thereby avoiding false positives that may arise from serological tests, which cannot exclude individuals who have recovered from HP infection. We have revised the Methods section (subsection: **HP detection**) to clarify the rationale for selecting the UBT.

Methods, 4) The DPB threshold of ≥4 for determining HP infection is mentioned but lacks a citation to validate its reliability.

**Response:** Thank you for pointing out this oversight. We have now included two citations to validate the DPB threshold of ≥4 for determining HP infection. This reference has been added to the Methods section.

Methods, 5) Although the FRS is a widely accepted tool for assessing CVD risk, it has limitations for diabetic populations, such as underestimating risk in this specific group. Using alternative tools, which are designed for people with diabetes, might have offered better risk stratification. Additionally, the thresholds for low, intermediate, and high risk in this study might not align with the higher baseline CVD risk in diabetic individuals. The authors should discuss whether these categorizations are valid for this population.

**Response:** We thank the reviewer for raising this important point regarding the limitations of the Framingham Risk Score (FRS) in diabetic populations and the validity of the risk thresholds used in this study. To address this concern, we have revised the Methods section to explicitly acknowledge the potential for FRS to underestimate CVD risk in diabetic populations due to their inherently higher baseline risk. We also discuss the availability of alternative tools, such as the UKPDS Risk Engine, which are specifically designed for diabetic populations. However, we chose the FRS for this study due to its widespread acceptance, ease of application, and comparability with other studies. Additionally, we have clarified that the thresholds for low (<10%), intermediate (10%-20%), and high (>20%) risk used in this study are consistent with prior research and have been widely validated across both general and specific populations, including diabetic cohorts. This point is supported by references to relevant studies (e.g., [29, 30, 32]) in the revised Methods section. The revised text now ensures that the limitations of the FRS and the rationale for its use, as well as the validity of the thresholds, are adequately addressed.

Methods, 6) The authors state that persons with "unreliable laboratory test results" were excluded. Please elaborate on what constitutes an "unreliable test result" in this study. Were there predefined criteria, such as hemolyzed samples or technical errors, or was this determined subjectively? Additionally, who defined and identified these cases, and how were they documented?

**Response:** We appreciate the reviewer’s request for clarification. We have now elaborated on what constitutes an “unreliable laboratory test result” in the Study Population section (Paragraph 2). Specifically, we have defined these as cases with hemolyzed samples as determined by the laboratory technicians. The criteria and procedures for documenting such cases have also been described.

Methods, 7) The authors mention the use of questionnaires to collect data on tobacco and alcohol consumption but do not specify which questionnaires were used. If these are standardized tools, proper citations should be provided. If they are modified or custom-made by the authors, the full questionnaires should be included as supplementary material to ensure transparency and reproducibility.

**Response:** We appreciate your insightful comment regarding the questionnaires used in our study. In response, we have revised the Methods section to specify that we developed a structured questionnaire for data collection on tobacco and alcohol consumption, as well as physical activity. This questionnaire was based on the World Health Organization's guidelines [23] and previous studies in the field [24]. Proper citations have already been added to acknowledge the sources that informed our questionnaire design.

Results, 1) For the tables, the authors may consider bolding significant p-values or using symbols such as asterisks to highlight them. This would help readers quickly identify statistically significant results, particularly given the large amount of data presented.

**Response:** We have now revised the manuscript by bolding all significant p-values in the tables (Tables 1–4). This enhancement will help readers quickly identify statistically significant results, especially given the large amount of data presented.

Results, 2) The authors state that “AST [which appears to be a typo and should likely be ALT!], AST, FBG, height, weight, and SBP were correlated with TG”, as well as other correlations in subsequent statements. Please clarify the direction of these correlations, were they positive or negative? Providing this detail is essential for accurately interpreting the relationships presented.

**Response:** Thank you for your valuable feedback on our manuscript. As per your suggestion, we have revised the paragraph to include the direction of the correlations found in our univariate analysis. Specifically, we have indicated whether the relationships between variables and lipid levels are positive or negative. This addition aims to enhance the clarity and understanding of our results.

Discussion, 1) The authors may overstate the implications of their findings by suggesting that HP infection is a risk factor for dyslipidemia and increased cardiovascular risk in the diabetic population. However, the cross-sectional design of the study limits causal inference. While this limitation is acknowledged in parts of the paper, it is downplayed in other areas, particularly toward the end of the Discussion, and even in the Conclusion. This acknowledgment should be emphasized consistently throughout the manuscript, and stronger language should be used when discussing causation—for example, "association observed" rather than "risk factor identified”, to avoid misleading the reader.

**Response:** Thank you for your careful reading of our manuscript and for your insightful comments. We appreciate your concern that we may have overstated the implications of our findings by suggesting that H. pylori (HP) infection is a risk factor for dyslipidemia and increased cardiovascular risk in the diabetic population. We agree that the cross-sectional design of our study limits causal inference and that this limitation should be consistently emphasized throughout the manuscript.

In response to your feedback, we have made the following revisions:

1. **Consistent Emphasis on Study Limitations:**

- Abstract: We have revised the conclusion to reflect the associative nature of our findings. The revised conclusion now reads: "H. pylori infection is associated with dyslipidemia and may contribute to an increased risk of cardiovascular disease in patients with diabetes."
- Discussion and Conclusion Sections: We have ensured that the limitations of our cross-sectional study are acknowledged consistently. Specifically, in the Conclusion of the Discussion section, we have added: "However, the cross-sectional nature of this study limits the ability to infer causation, and further longitudinal studies are warranted to confirm these findings."

2. **Use of Appropriate Language to Avoid Causal Inference:**

Throughout the manuscript, we have replaced definitive terms such as "risk factor" with language that more accurately reflects association rather than causation. For example:

- In the Discussion, we altered statements to read: "Even after adjusting for all factors affecting lipid metabolism in the logistic regression model, infection with HP was independently associated with lower HDL-C and higher TC and LDL-C levels in patients with diabetes."
- We changed "HP may be a risk factor for increased risk of dyslipidemia and cardiovascular disease in patients with diabetes" to: "We observed that HP infection may be associated with an increased risk of dyslipidemia and cardiovascular disease in patients with diabetes, consistent with previous findings [36, 37]."

3. **Strengthening Statements on Causation:**

- We have reinforced the acknowledgment of our study's limitations in the Discussion: "As a cross-sectional investigation, it can only demonstrate the correlation between HP infection and abnormalities of lipid metabolism and cannot clarify the causation between them. This research is insufficient to determine whether eradicating HP would improve lipid metabolism and reduce the risk of cardiovascular disease in patients with diabetes."
- We emphasize the need for future studies: "Therefore, future studies should compare changes in lipid levels and cardiovascular disease risk before and after HP eradication treatment in individuals with diabetes."

4. **Consistent Terminology:** We have ensured that terms implying causation are avoided throughout the manuscript. Phrases like "HP infection is an independent risk factor" have been adjusted to "HP infection is independently associated with."

We believe these revisions appropriately address your concerns and ensure that our conclusions accurately reflect the limitations of our study design. Your feedback has been invaluable in refining our manuscript to prevent potential misinterpretation by readers.

Discussion, 2) The authors propose multiple mechanisms linking HP to dyslipidemia, such as altered gut microbiota and liver dysfunction. While these are plausible, none of these pathways are directly investigated in this study, which makes these explanations speculative. The authors should clearly state that these mechanisms are hypothetical and recommend that future studies explore them in detail to validate these claims.

**Response:** We thank the reviewer for pointing out that our discussion of the mechanisms is speculative and needs clarification. In the revised Discussion, we have now explicitly stated that the proposed pathways by which HP may contribute to dyslipidemia—via inflammatory responses, hepatic dysfunction, and gut microbiota alterations—were **not** directly investigated in this study and, therefore, remain **hypothetical**. Specifically, we have added the following text:

“*Although these mechanisms are biologically plausible, it is important to emphasize that we did not investigate any of these pathways directly in the present study. Consequently, the proposed explanations for how HP may affect lipid profiles remain speculative based on the existing literature. We recommend that future studies explore these pathways in depth—potentially through mechanistic and longitudinal research designs—in order to validate the hypotheses regarding HP-induced inflammatory, hepatic, and gut microbiota changes that might contribute to dyslipidemia in patients with diabetes.*”

With this modification, we believe we have addressed the reviewer’s concern by clearly acknowledging that these mechanisms are theoretical in nature within the context of our current data, and by **highlighting the need for future mechanistic studies** to fully elucidate the role of HP in dyslipidemia and cardiovascular risk among patients with diabetes.

Discussion 3) Inconsistencies in the use of abbreviations and short forms are noticeable throughout the manuscript. Please revise the text to ensure consistency. For example, HDL and LDL are used multiple times before being reintroduced as "high-density lipoprotein" and "low-density lipoprotein” later in the text. Similarly, "HP" is redefined as Helicobacter pylori in the Discussion after numerous uses of HP throughout the text.

**Response:** We apologize for the inconsistent use of abbreviations in the manuscript. We have thoroughly reviewed the text to ensure that all abbreviations are properly defined upon their first mention and consistently used thereafter. Specifically, terms such as HDL, LDL, and HP have been revised to maintain clarity and uniformity throughout the manuscript.

Reviewer #5: This is an association study carried out on retrospective data and the ethical approval has been waived due to retrospective data collection. However, it seems some data collection has been done prospectively.
Such studies have week power for evidence generation. As authors have mentioned in the 'Limitation of the Study', a stronger evidence could have been produced by observing lipid parameters in two groups of H pylori infection divided on the basis of evidence-based treatment and placebo. Such a study may get a space in a Q1 journal like PLOS ONE.

**Response:**

We appreciate the reviewer’s insightful comments and constructive feedback. Below, we address each point raised:

1. Retrospective vs. Prospective Data
   - We confirm that all data used in our analysis were obtained from archived medical examination records (January 1 to May 31, 2024) at the Health Management Center, as stated in the Methods (“Study Population”). No new prospective recruitment or follow-up occurred; instead, we utilized existing routine health check data. The study was approved by the Ethics Committee of Deyang People's Hospital (Ethical review approval number: LWH-OP-006-A04-V2.0), and the requirement for informed consent was waived due to the retrospective nature of the study.
   - We acknowledge that retrospective data inherently limit the ability to infer causality, which is why we classify our study as cross-sectional and have highlighted this in our Discussion and “Limitations of the Study.”
2. Study Design and Evidence Generation
   - We agree that a randomized controlled trial or longitudinal design—monitoring patients before and after H. pylori eradication—could produce more robust evidence of causality. In our Discussion section, we explicitly note that our study can only show associations, not confirm causality.
   - As the reviewer correctly points out, such a design (comparing H. pylori-eradicated vs. placebo) would strengthen evidence quality; we therefore emphasize in the “Limitations” that future prospective or interventional studies are warranted to validate our findings and clarify the causal relationships.
3. Study Value and Future Directions
   - Despite its cross-sectional nature, this study provides preliminary insights into the possible link between H. pylori infection and dyslipidemia in individuals with type 2 diabetes.
   - We will certainly consider the reviewer’s advice for more rigorous prospective or interventional designs in future research to further elucidate the mechanistic pathways and causality of H. pylori-related dyslipidemia.

We appreciate the reviewer’s constructive feedback and believe our revisions and clarifications address the concern regarding the retrospective design and limited power for causal inference.

6. PLOS authors have the option to publish the peer review history of their article (what does this mean?). If published, this will include your full peer review and any attached files.


If you choose “no”, your identity will remain anonymous but your review may still be made public.


**Do you want your identity to be public for this peer review?** For information about this choice, including consent withdrawal, please see our Privacy Policy.

Reviewer #1: No

Reviewer #2: No

Reviewer #3: No

Reviewer #4: **Yes:** Afshin Heidari

Reviewer #5: No
